# Supplementary material for: Crowding-induced opening of the mechanosensitive Piezo1 channel in silico
Source: Commun Biol. 2021 Jan 19;4:84. doi: 10.1038/s42003-020-01600-1 (PMC7815867; doi:10.1038/s42003-020-01600-1)
Supplement: Supplementary file 2 — Supplementary Information [file 42003_2020_1600_MOESM2_ESM.pdf]

Crowding-induced Opening of the Mechanosensitive Piezo1 Channel in Silico

Wenjuan Jiang[1], John Smith Del Rosario[2$], Wesley Botello-Smith[1$], Siyuan Zhao[2], Yi-chun Lin[1], Han Zhang[1], Jérôme Lacroix[3]*, Tibor Rohacs[2]*, Yun Lyna Luo[1]*

SUPPLEMENTARY MATERIALS

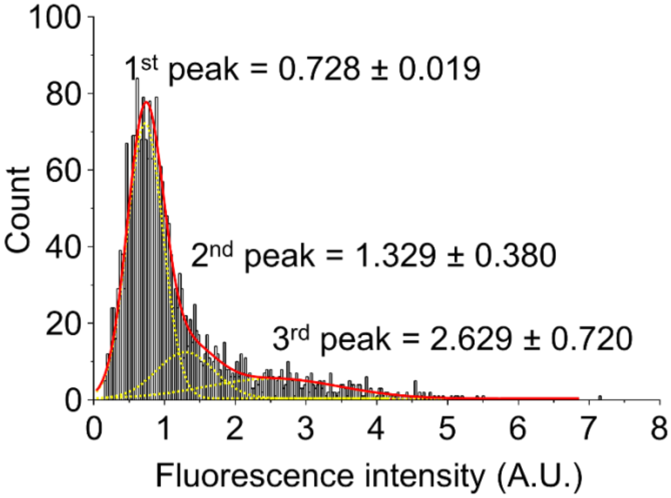

Figure S1. Distribution of fluorescence intensity of GFP-mPiezo1 puncta observed by TIRF in HEK293 cells. The plot shows the distribution of fluorescence intensity obtained from 2398 eGFP-Piezo1 randomly selected puncta from 28 cells. The TIRF illumination power and exposure time were identical for all cells. The profile is well fitted (R^2=0.9668) with a 3-gaussian function with a major peak (0.728 ± 0.019) and two minors peaks (1.329 ± 0.380 and 2.629 ± 0.720).

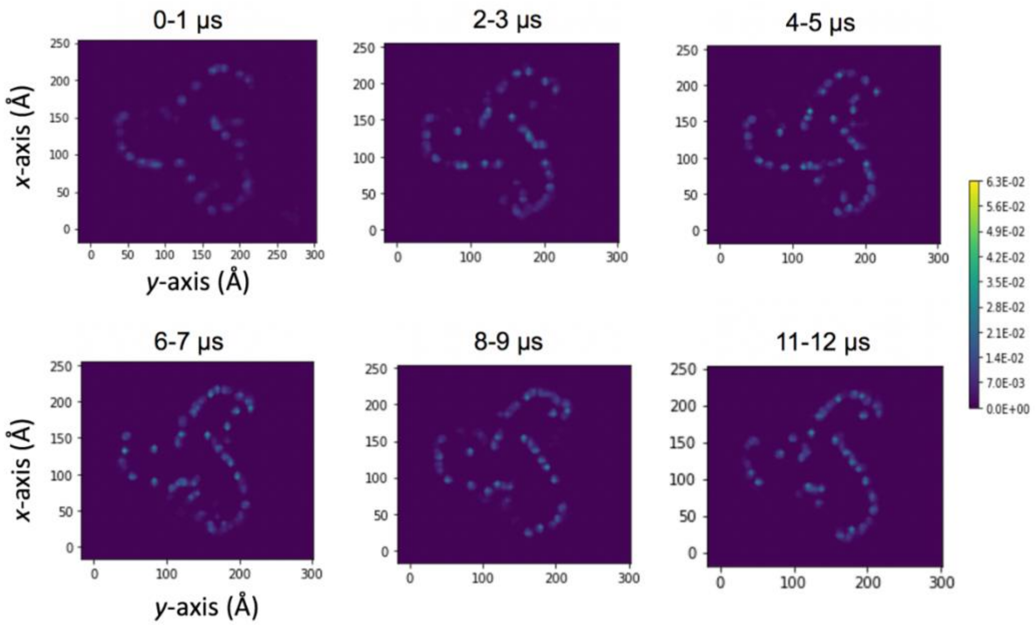

Figure S2: 2D lateral density maps of PIP_2 at lower leaflet over CG simulation trajectory.

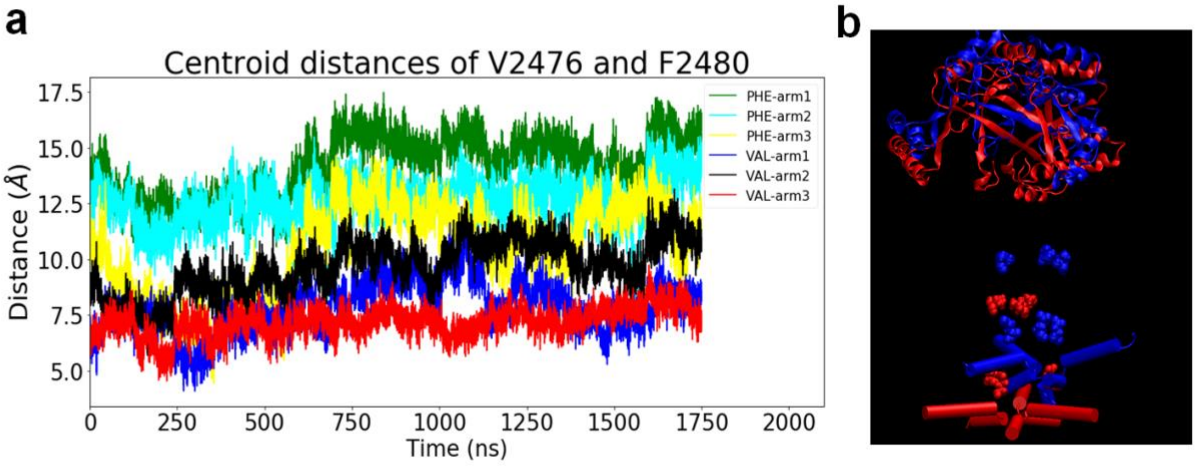

Figure S3: a. Centroid distances of three F2480 and three V2476 during 1.75 μs MD simulation (*box 1* system). The centroid distance of F2480 is calculated from the triangle formed by sidechain carbon atom type CZ of F2480 residues from three subunits. **b.** The cap and CTD positions before (red) and after 2 μs simulation (blue), indicating the CTD moved upward upon Piezo1 arm flattening (*box1* system). Residue V2476 and E2495 are shown in VDW.

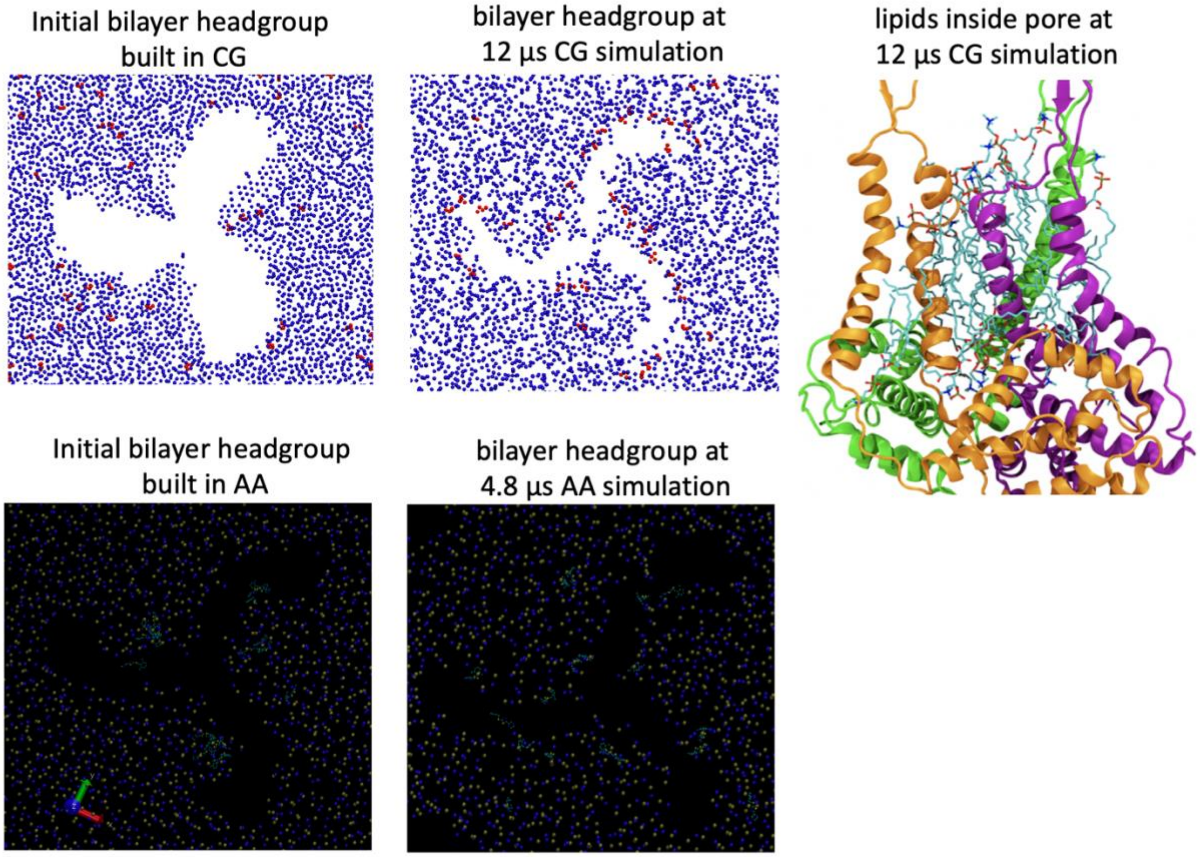

Figure S4: Lipid headgroups at the beginning and end of the CG and AA simulations. The AA data are taken from Ref 21. In CG, blue dots are POPC headgroups, and red dots are PIP2 headgroups. In AA, blue dots are nitrogen atoms, yellow dots are phosphorus atoms in POPC headgroups, Yoda1 molecules are shown in cyan licorice. On the right, lipids are shown in licorice with atom color code (red oxygen, blue nitrogen, cyan carbon, tan phosphorus). The backbone of Piezo1 pore is shown in newcartoon mode with different colors for each subunit (orange, green, and purple).

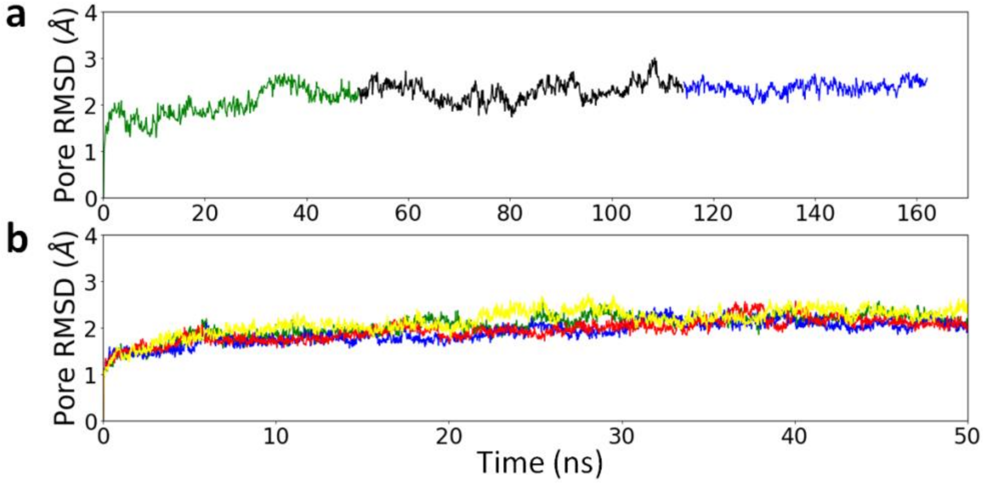

Figure S5: a) Root-mean-square deviation (RMSD) of inner pore helices backbone of Piezo1 under different membrane tensions without voltage. Lateral pressure of 10, 8, 6, 4, 2 bars correspond to 14.2, 11.4, 8.5, 5.7, 2.8 mN/m. The green line stands for 50ns with 10bar, the black line stands for 64 ns with lateral pressure from 8 bar to 2 bar, each tension runs for 16 ns, and the blue line stands for 48ns with 0 bar **b**) RMSD of inner pore helices backbone under different voltages: -500mV in green, -250mV in blue, 250mV in red and 500mV in yellow lines.

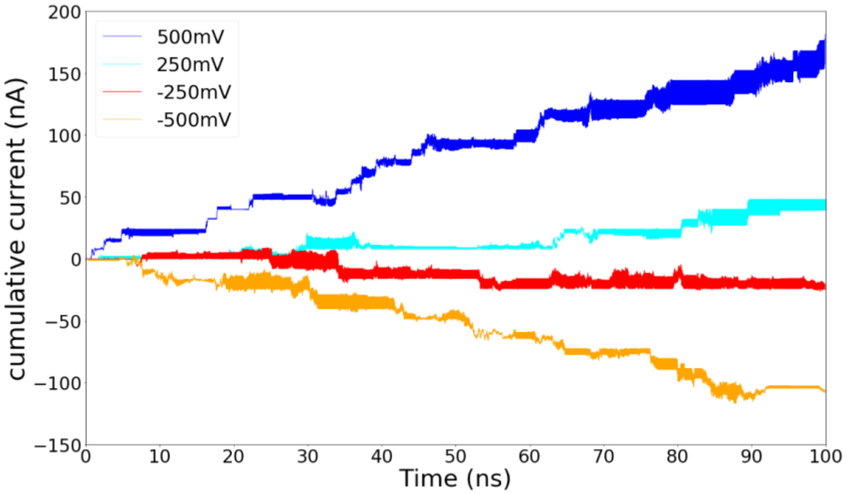

Figure S6: Cumulative currents though the inner pore helices of Piezo1 under different voltages over two replicas (in total 100ns). The current is calculated from Approach 2, referring as “z-displacement method”.

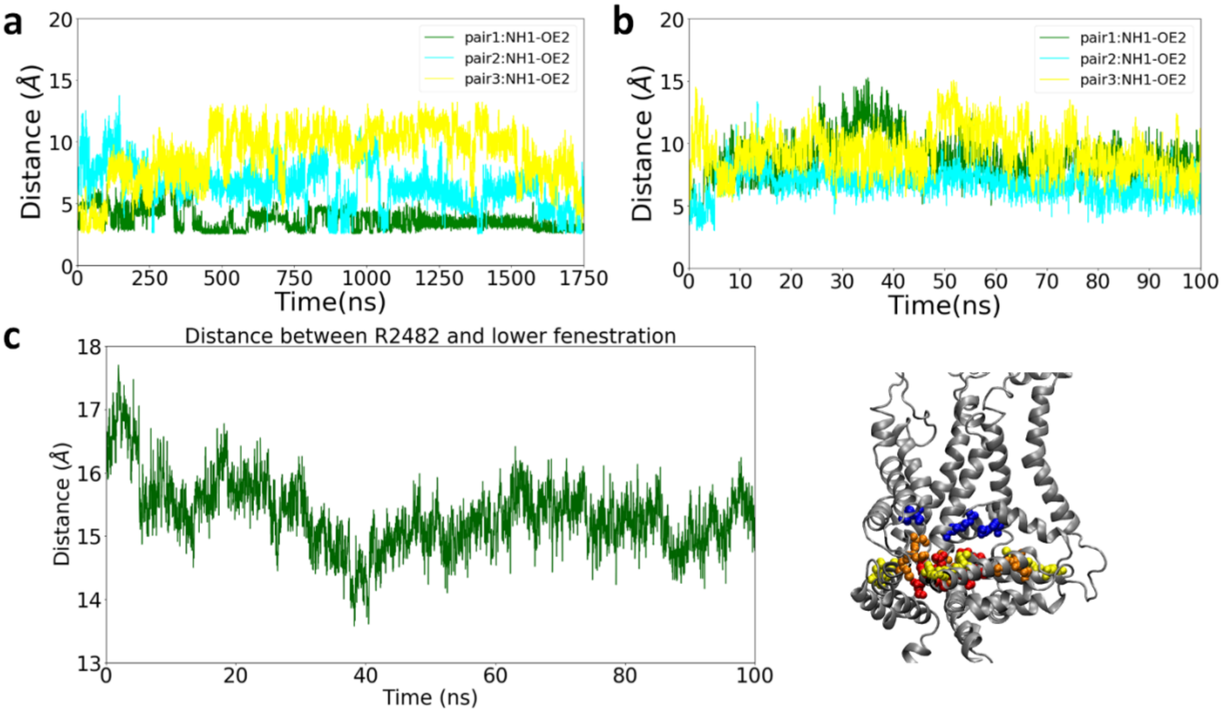

Figure S7. Saltbridge distance between sidechain oxygen of E2133 and sidechain nitrogen of R2482 for WT Piezo1 over 1.75μs in *box1* system (**a**) and E2133Q mutant mimic over 100ns (**b**). **c**) Distance between R2482 sidechain and the center of the lower fenestration averaged over three subunits. The center of each lower fenestration is defined by the total center of mass of nine residues in the fenestration C2154, S2150, N2151, S2491, E2495/6, I2164, S2168, E2171. Figure on the right illustration the position of R2482 above the lower fenestration. Color scheme: R2482 in blue; C2154, S2150, N2151 in orange; S2491, E2495/6 in red; I2164, S2168, E2171 in yellow. Pore helix and CTD regions are shown in grey in newcartoon.

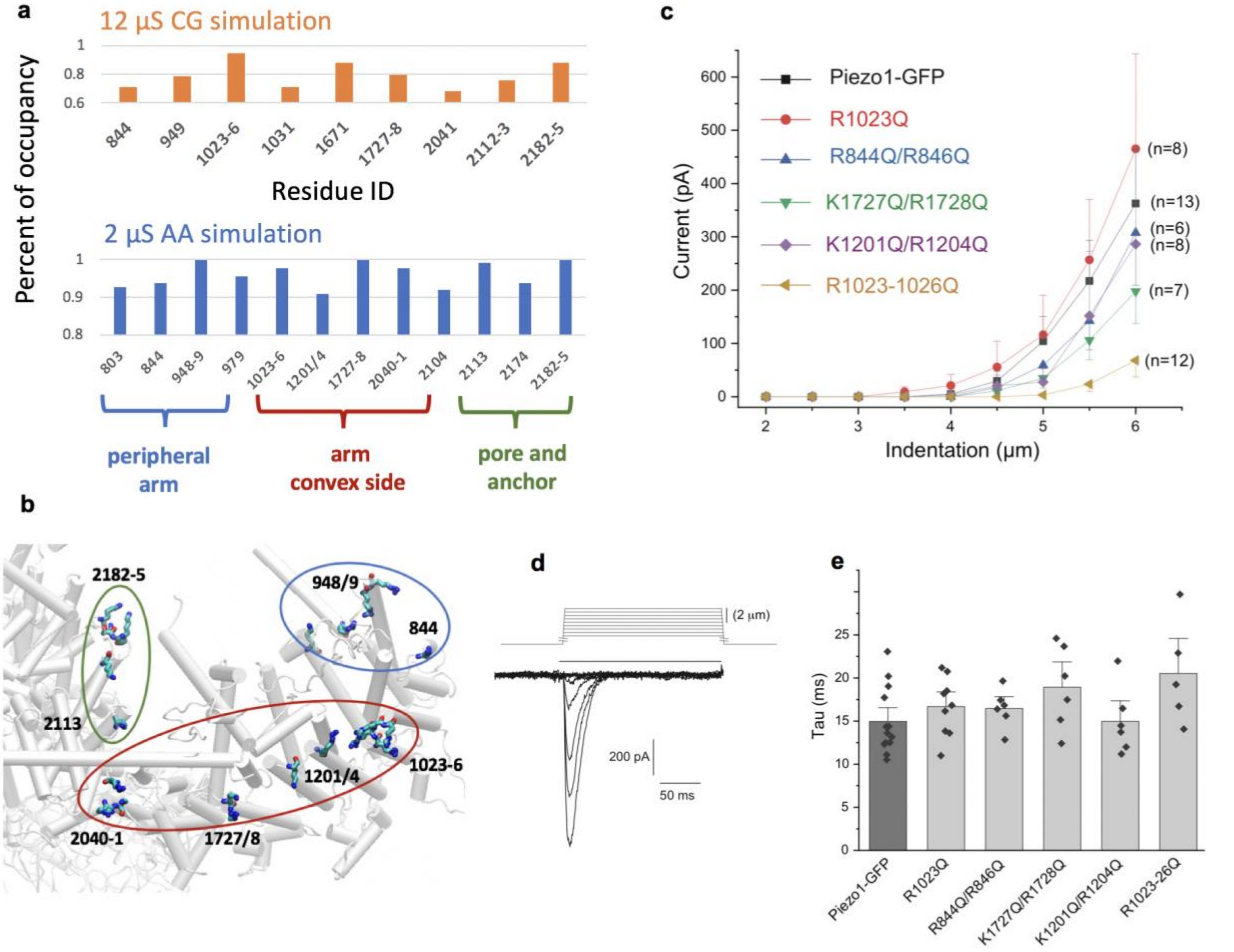

Figure S8. The effect of mutating PIP_2 interacting residue clusters on Piezo1 channel activity. (**a)** The residues in mouse Piezo1 that have maximum PIP_2 occupancy above 60% of 12 µs CG simulation or above 90% of 2 µs AA *box1* simulation. (**b)** Hotspot residues clustered by locations on the Piezo1 structure (bottom view). (**c)** Summary of current amplitudes in response to increasing mechanical stimuli for wild type and mutant Piezo1 channels. Whole cell patch clamp experiments on HEK293 cells transfected with the GFP-tagged mouse Piezo1 and its mutants were performed as described in the methods section. Data are shown as mean +/- SEM, the number of cells tested are indicated for each mutant. (**d)** Representative trace of wild type Piezo1 currents in response to increasing mechanical indentations. **(e)** Summary of the inactivation time constant for wild type and mutant Piezo1 currents. Data are shown as mean +/- SEM, and scatter plots.

Table S1. Details of CG and AA Piezo1 simulation systems.

System		Inner		Outer	no. of		
		PC	PIP2	PC	Water	Na^+	Cl^+
CG	POPC : POPC/PIP_2	831	41	951	109414	1252	1157
AA	POPC : POPC/PIP_2	587	39	664	185688	799	712

Table S2. Details of extended all-atom Piezo1 simulation systems.

System	Inner		Outer	Number of			x-dim (nm)	y-dim (nm)	xy area (nm^2)	%Protein Occupancy*		Time (μs)
	POPC	PIP_2	POPC	Water	K^+	Cl^-				Inner	Outer	
**Box1**	537	38	607	160133	695	612	19.3	25.1	484.4	19.0	14.4	2.00
**Box2**	534	38	610	161136	721	638	19.9	24.4	485.5	19.6	14.2	1.75
**Box3**	519	37	590	151351	753	674	21.3	22.5	479.2	20.8	15.9	1.75

***** Protein occupied area is estimated by subtracting the lipid surface area from the total PBC box xy area for upper and lower leaflet individually. The area per lipid reported in Charmm36 force field is 68.3$Å^2$ per POPC and 67.4$Å^2$ per PIP_2:

$$Occupancy_{UpperLeaflet} = \frac{BoxArea_{XY} - N._{POPC} * Area_{POPC}}{BoxArea_{XY}}$$

$$Occupancy_{LowerLeaflet} = \frac{BoxArea_{XY} - N._{POPC} * Area_{POPC} - N._{PIP2} * Area_{PIP2}}{BoxArea_{XY}}$$

Table S3. Piezo dome-dome distance (R) in nm along *x* and *y* axis (see Figure 2b). The average dome radius (r) is 9.2 nm.

	Box1	Box2	Box3
x-dim	19.3	19.9	21.3
R along *x*	0.9	1.5	2.9
y-dim	25.1	24.4	22.5
R along *y*	6.7	6.0	4.1

Table S4. Number of permeation events for WT and mutant Piezo1 channels using two approaches*.

System	Voltage (mV)	Time (ns)	K^+ / 100ns		Cl^- /100ns		Conductance (pS)		K^+ / Cl^- ratio	
			Approach 1	Approach 2	Approach 1	Approach 2	Approach 1	Approach 2	Approach 1	Approach 2
**WT**	500	100	11 (6.9-16.5)	13±2.2	7 (3.8-11.5)	10±2.3	39.7 (22.4-66.8)	52.0±32.2	2.9	2.5
**WT**	250	100	3 (1.1-6.2)	4±1.8	2 (0.6-4.8)	2±1.9				
**WT**	-250	100	3 (1.1-6.2)	3±1.9	0 (0-1.0)	0±2.4				
**WT**	-500	100	9 (5.3-14.0)	12±1.7	0 (0-1.0)	1±1.5				
**E2133Q**	500	100	1 (0.5-3.2)	6±2.8	7 (3.8-11.5)	14±2.4	N/A		0.1	0.4
**9K-muta**	500	100	1 (0.5-3.2)	10±1.1	9 (5.3-14.0)	19±2.1			0.1	0.5

*****Approach 1 is the boundary-crossing approach (see Methods). The uncertainty in parentheses is 85% confidence interval based on a Poisson distribution. Approach 2 is the z-displacement approach. The standard deviation is estimated from bootstrapping.

Table S5. GROMACS topology file (bead type) for Martini PI(4,5)P_2 model*.

nr	type	resnr	residue	atom	cgnr	charge
1	P1	1	POP5	C1	1	0
2	Na	1	POP5	C2	2	0
3	P4	1	POP5	C3	3	0
4	Qa	1	POP5	PO4	4	-1.0
5	Qa	1	POP5	P1	5	-2.0
6	Qa	1	POP5	P2	6	-1.0
7	Na	1	POP5	GL1	7	0
8	Na	1	POP5	GL2	8	0
9	C1	1	POP5	C1A	9	0
10	C4	1	POP5	D2A	10	0
11	C4	1	POP5	D3A	11	0
12	C1	1	POP5	C4A	12	0
13	C1	1	POP5	C1B	13	0
14	C1	1	POP5	C2B	14	0
15	C1	1	POP5	C3B	15	0
16	C1	1	POP5	C4B	16	0

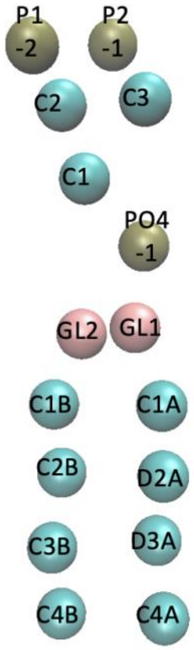

*The parameter file itp and pdb, as well as a modified INSANE script are provided at https://github.com/reneejiang/cg_pip2_topology_files

Table S6. GROMACS topology file (bone length, angles, constraints) for Martini PI(4,5)P_2 model

Bond length					Angles						Constraints			
**i**	**j**	**funct**	**length**	**force**	**i**	**j**	**k**	**funct**	**angle**	**force**	**i**	**j**	**funct**	**length**
1	2	1	0.40	30000	1	4	7	2	140.0	25.0	1	2	1	0.40
1	3	1	0.40	30000	7	9	10	2	180.0	25.0	1	3	1	0.40
2	3	1	0.40	30000	9	10	11	2	100.0	10.0	2	3	1	0.40
2	5	1	0.30	25000	10	11	12	2	120.0	45.0	2	5	1	0.30
2	6	1	0.35	30000	8	13	14	2	180.0	25.0	2	6	1	0.35
1	5	1	0.40	25000	13	14	15	2	180.0	25.0	1	5	1	0.40
3	6	1	0.31	30000	14	15	16	2	180.0	25.0	3	6	1	0.31
5	6	1	0.60	25000										
1	4	1	0.35	1250										
4	7	1	0.47	1250										
7	8	1	0.37	1250										
7	9	1	0.47	1250										
9	10	1	0.47	1250										
10	11	1	0.47	1250										
11	12	1	0.47	1250										
8	13	1	0.47	1250										
13	14	1	0.47	1250										
14	15	1	0.47	1250										
15	16	1	0.47	1250										

Table S7. Bond length comparison between CG and AA model for PI(4,5)P_2

Bond Pairs	Atom Model(nm)	CG model (nm)	Difference (nm)	%Diff
C1_C2	0.27	0.40	0.13	46.82
C1_C3	0.32	0.40	0.08	24.84
C2_C3	0.27	0.40	0.13	46.02
C2_P1	0.33	0.30	-0.03	-9.41
C2_P2	0.33	0.35	0.02	5.69
C1_P1	0.44	0.40	-0.04	-8.64
C3_P2	0.43	0.31	-0.12	-28.11
P1_P2	0.61	0.60	-0.01	-1.03
C1_PO4	0.34	0.31	-0.02	-6.08
PO4_GL1	0.43	0.45	0.02	3.34
GL1_GL2	0.27	0.35	0.08	29.61
GL1_C1A	0.51	0.46	-0.05	-9.90
C1A_D2A	0.44	0.45	0.01	2.14
D2A_D3A	0.52	0.45	-0.07	-13.34
D3A_C4A	0.52	0.45	-0.06	-12.11
GL2_C1B	0.63	0.45	-0.18	-28.22
C1B_C2B	0.52	0.45	-0.07	-14.09
C2B_C3B	0.47	0.45	-0.03	-5.30
C3B_C4B	0.47	0.45	-0.02	-4.01

Table S8. Angle comparison between CG and AA model for PI(4,5)P_2

Angle Pairs	Atom Model (degrees)	CG model (degrees)	Difference (degrees)	%Diff
C1_PO4_GL1	101.35	132.26	30.92	30.51
GL1_C1A_D2A	107.09	141.37	34.28	32.01
C1A_D2A_D3A	109.43	92.07	-17.37	-15.87
D2A_D3A_C4A	105.27	118.42	13.15	12.49
GL2_C1B_C2B	110.94	138.13	27.19	24.51
C1B_C2B_C3B	138.20	136.60	-1.61	-1.16
C2B_C3B_C4B	134.62	137.64	3.02	2.24

Table S9. Radius of gyration comparison between CG and AA model for PI(4,5)P_2

PI(4,5)P_2	Atom model	CG model	Difference	%Diff
Radius of Gyration(nm)	0.77	0.81	0.04	5.28
