# Supplementary material for: Crowding-induced opening of the mechanosensitive Piezo1 channel in silico
Source: Commun Biol. 2021 Jan 19;4:84. doi: 10.1038/s42003-020-01600-1 (PMC7815867; doi:10.1038/s42003-020-01600-1)
Supplement: Supplementary file 3 — Description of Additional Supplementary Files [file 42003_2020_1600_MOESM3_ESM.pdf]

**Description of Additional Supplementary Files**

**File name:** Supplementary Movie 1.
**Description: A single potassium permeation event.** Trajectory of a permeating $K^+$ ion during 24 ns simulation under -500 mV voltage. The backbone of the Piezo1 cap and pore domain is shown in orange. The DEEED residues are shown in licorice with the atom color code (red oxygen, blue nitrogen, cyan carbon).

**File name: Supplementary Data 1.**
**Description:** Source data for Figure 2-6 and Supporting tables in Excel format. Source data in Figure 1 is provided in the python code.
