# Supplementary material for: Crowding-induced opening of the mechanosensitive Piezo1 channel in silico
Source: Commun Biol. 2021 Jan 19;4:84. doi: 10.1038/s42003-020-01600-1 (PMC7815867; doi:10.1038/s42003-020-01600-1)
Supplement: Supplementary file 6 — Reporting Summary [file 42003_2020_1600_MOESM6_ESM.pdf]

nature research

Corresponding author(s): Yun Lyna Luo

Last updated by author(s): Dec 10, 2020

Reporting Summary

Nature Research wishes to improve the reproducibility of the work that we publish. This form provides structure for consistency and transparency in reporting. For further information on Nature Research policies, see Authors & Referees and the Editorial Policy Checklist.

Statistics

For all statistical analyses, confirm that the following items are present in the figure legend, table legend, main text, or Methods section.

n/a	Confirmed	
☐	☒	The exact sample size (*n*) for each experimental group/condition, given as a discrete number and unit of measurement
☐	☒	A statement on whether measurements were taken from distinct samples or whether the same sample was measured repeatedly
☐	☒	The statistical test(s) used AND whether they are one- or two-sided<br>*Only common tests should be described solely by name; describe more complex techniques in the Methods section.*
☒	☐	A description of all covariates tested
☒	☐	A description of any assumptions or corrections, such as tests of normality and adjustment for multiple comparisons
☐	☒	A full description of the statistical parameters including central tendency (e.g. means) or other basic estimates (e.g. regression coefficient) AND variation (e.g. standard deviation) or associated estimates of uncertainty (e.g. confidence intervals)
☒	☐	For null hypothesis testing, the test statistic (e.g. *F*, *t*, *r*) with confidence intervals, effect sizes, degrees of freedom and *P* value noted<br>*Give P values as exact values whenever suitable.*
☒	☐	For Bayesian analysis, information on the choice of priors and Markov chain Monte Carlo settings
☒	☐	For hierarchical and complex designs, identification of the appropriate level for tests and full reporting of outcomes
☒	☐	Estimates of effect sizes (e.g. Cohen's *d*, Pearson's *r*), indicating how they were calculated

Our web collection on statistics for biologists contains articles on many of the points above.

Software and code

Policy information about availability of computer code

Data collection	CG MD simulations were conducted using GROMACS version 2016.4. AA MD simulations were conducted using pmemd.cuda version of AMBER18 and a specialized MD engine on Anton2 supercomputer.
Data analysis	MD data analysis were conducted using VMD1.9.3 and AmberTools 18 version of CPPTRAJ, and in-house python scripts below.<br>Python3 code for hyperbolic tangent mode is available for download at<br>https://github.com/wesleymsmith/MembraneFootprintInteractionModel<br>Python3 code for performing Gaussian 2D model fitting of anisotropic membrane curvature is available for download at https://<br>github.com/wesleymsmith/Piezo_Membrane_Gauss_2D_Model

For manuscripts utilizing custom algorithms or software that are central to the research but not yet described in published literature, software must be made available to editors/reviewers. We strongly encourage code deposition in a community repository (e.g. GitHub). See the Nature Research guidelines for submitting code & software for further information.

Data

Policy information about availability of data

All manuscripts must include a data availability statement. This statement should provide the following information, where applicable:

- Accession codes, unique identifiers, or web links for publicly available datasets
- A list of figures that have associated raw data
- A description of any restrictions on data availability

All simulation input/output files and trajectories are publicly available on Anton2 supercomputer.
Source data underlying figures is provided in Supplementary Data 1.
Re-parameterized PI(4,5)P2 Martini CG model is available for download at https://github.com/reneejiang/cg_pip2_topology_files

Field-specific reporting

Please select the one below that is the best fit for your research. If you are not sure, read the appropriate sections before making your selection.

☒ Life sciences ☐ Behavioural & social sciences ☐ Ecological, evolutionary & environmental sciences

For a reference copy of the document with all sections, see nature.com/documents/nr-reporting-summary-flat.pdf

Life sciences study design

All studies must disclose on these points even when the disclosure is negative.

Sample size	No sample-size calculation was performed. Sample sizes were similar to those in the literature
Data exclusions	All data were included
Replication	All replication attempts were successful, experiments were performed on cells from at least 3 different transfections for each condition.
Randomization	Experiments were performed in a random order on the different mutants
Blinding	Data collection was not blinded, as this is highly impractical and not usual in electrophysiology experiments

Reporting for specific materials, systems and methods

We require information from authors about some types of materials, experimental systems and methods used in many studies. Here, indicate whether each material, system or method listed is relevant to your study. If you are not sure if a list item applies to your research, read the appropriate section before selecting a response.

Materials & experimental systems

n/a	Involved in the study	
☒	☐	Antibodies
☐	☒	Eukaryotic cell lines
☒	☐	Palaeontology
☒	☐	Animals and other organisms
☒	☐	Human research participants
☒	☐	Clinical data

Methods

n/a	Involved in the study	
☒	☐	ChIP-seq
☒	☐	Flow cytometry
☒	☐	MRI-based neuroimaging

Eukaryotic cell lines

Policy information about cell lines

Cell line source(s)	This is stated in the methods section
Authentication	The cell line was authenticated by ATCC, and we use low passage number cells
Mycoplasma contamination	Cells were free of mycoplasma
Commonly misidentified lines (See ICLAC register)	N/A
